# Discovering mythorealism: A corpus stylistic analysis of Yan Lianke’s novels in English

**DOI:** 10.1371/journal.pone.0342696

**Published:** 2026-02-24

**Authors:** Yi Zhang, Chaoyong Zhao

**Affiliations:** School of Foreign Languages, East China Normal University, Shanghai, China; Bahir Dar University, ETHIOPIA

## Abstract

This study aims to identify the stylistic features of the literary mode Yan Lianke terms as “mythorealism” in the English translations of his novels, through corpus stylistic analysis. Using a corpus of nine translated works by Yan and a reference corpus of English translations of contemporary Chinese fiction, the analysis employs Wmatrix to detect statistically overused semantic domains and LancsBox to investigate their collocational networks and usage contexts. Combining quantitative and qualitative methods, the study identifies five foregrounded semantic patterns: political discourse, spatial narrative, natural environment, color symbolism and the supernatural. The findings show that political discourse intertwines historical events with allegorical critique; spatial narrative delineates symbolic boundaries between social and psychological worlds; color symbolism, particularly the recurrent use of red, conveys culturally resonant yet ambivalent meanings; and supernatural elements extend realism into metaphysical and philosophical realms. Supported by a representative bilingual case study which illustrates the retention of core semantic structures, the study suggests that despite translator mediation, these patterns largely reflect the enduring thematic and stylistic characteristics of Yan’s fiction. These findings offer a corpus-based empirical grounding for mythorealism and present a replicable framework for bridging semantic-domain statistics and stylistic interpretation in the study of translated literature.

## 1. Introduction

Yan Lianke, one of China’s most celebrated yet controversial authors, has been recognized both for his imaginative satire [1] and his frequent encounters with censorship [2]. His narratives skillfully blur the lines between societal norms and political censorship, reflecting the complexities of modern China. Yan’s remarkable literary achievements include being the first Chinese recipient of the Kafka Prize for Literature, alongside receiving multiple accolades such as the Lu Xun Literary Awards, the Lao She Literary Award and the Japan Twitter Literary Award. Yan’s formative years were profoundly shaped by political upheavals, rural poverty, and pervasive corruption. These experiences have profoundly impacted his literary pursuits [3], inspiring works that often feature intense suffering and political themes, leading to their frequent bans in China.

While Yan’s literary career has garnered both acclaim and controversy [4–8], his novels have achieved significant international recognition. Although frequently associated with magical realism [9,10], Yan defines his distinct aesthetic as *shenshizhuyi* (神实主义), or “mythorealism.” His literary philosophy rests on two principal ideas. First, he positions himself as a rebellious successor to the traditional realism, famously declaring himself “realism’s unfilial son” [11]. Second, mythorealism diverges from conventional realism’s adherence to empirical cause-and-effect. Instead, it operates on “inner causality” (*nei yinguo* 内因果), a logic governed by the internal trajectories of the human psyche rather than external events. By abandoning the external causal logic typical of traditional and magical realism, mythorealism reconstructs reality governed by the “inner logic” of the human soul to excavate the “invisible truth,” the complex depths of Chinese historical reality that defy conventional explanation [11]. Expanding on this framework, Zheng and Niu [12] identify two defining features: the inversion of the individual-society dynamic, wherein the individual illuminates society rather than the reverse; and the bridging of reality and narrative through imaginative constructs rooted in the human soul, spirit and creativity.

Grounded in the concept of mythorealism, Yan’s novels chronicle over half a century of China’s revolutionary history and rural transformation, offering profound insights into the lived experiences of rural residents within their socio-historical contexts. Despite growing scholarly interest, existing research remains predominantly qualitative [3,13], leaving his stylistic features largely unexamined through the lens of large-scale or computational analysis. To address this gap, this study employs a corpus stylistic approach to investigate Yan’s fiction via its English translations. The decision is driven by a critical methodological necessity arising from the asymmetry of computational tools. While English semantic tagging systems are highly optimized, automated annotation for Chinese remains constrained by lower accuracy and granularity [14]. Hence, English translations serve as a vital methodological bridge, facilitating the retrieval of semantic macrostructures that might otherwise remain inaccessible. Aligning with Moretti’s [15] notion of “distant reading,” this approach allows for a systematic examination of Yan’s entire oeuvre, revealing recurrent stylistic and semantic patterns difficult to verify through qualitative interpretation alone [16]. By analyzing the linguistic construction of Yan’s narratives in English, the study aims to elucidate how “mythorealism” is stylistically constituted for a global readership.

## 2. Related work

### 2.1 Studies on Yan Lianke’s works

Yan Lianke’s novels, distinguished by their literariness, spirituality, and embrace of the absurd, have garnered a global readership and considerable scholarly interest. Research on Yan’s oeuvre typically falls into three thematic categories: mythorealism and censorship, literariness and literary creativity and translation and dissemination.

The first category examines the interaction between Yan Lianke’s narrative style and the socio-political landscape of contemporary China, particularly how the censorship apparatus influences his creation of mythorealistic narratives [10,17,18]. The second category focuses on the elements of absurdity, spirituality and creativity within his literary works, attracting considerable scholarly analyses [3,5,6,19]. For instance, Pesaro [20] identifies that Yan’s early fictions present clear indications of a new aesthetic, characterized by modernist techniques and strategies of disorientation. As for the third category, motivated by Yan’s innovative literary approach across historical, gender and cultural dimensions, it explores how his works are translated and received in various regions, including European countries like France and the Czech Republic, English-speaking countries and Southeast Asia, such as Japan and Thailand [21–23].

Existing research on Yan Lianke’s works has adopted a multi-perspective, multi-dimensional approach, thoroughly examining both the internal elements and external influences on his literary texts, offering incisive insights into his works. However, despite the rich qualitative analysis prevalent in these studies, there is a noticeable gap in corpus-based analyses that comprehensively cover his entire literary output. This study seeks to address this deficiency by employing corpus stylistic methods to systematically explore the distinctive features of Yan’s mythorealism.

### 2.2 Corpus stylistic and foregrounding

Since the 1980s, the emergence and accessibility of “big data” have propelled the growth of digital literary studies, marking a significant “corpus turn” within the field of literary analysis [24]. This development has redefined traditional literary studies, particularly by expanding the scope and methodology of data analysis. One key outcome of the shift is the rise of corpus stylistics, which applies corpus linguistics methods to literary texts for interpretive purposes, merging computational technologies with literary criticism [25,26]. It allows for a wider range of textual data to be analyzed, from complete works of individual authors to literary periods spanning several decades. Moreover, the use of computational corpus tools has refined research methodologies, enabling scholars to detect linguistic features, stylistic variations and intertextual and contextual influences. The data-driven approach has introduced a revisionist spirit, reinvigorating the field with fresh perspectives and methodologies [27].

In this study, corpus stylistics provides a methodological framework to systematically explore the distinctive features of Yan’s mythorealism. Central to this exploration is the concept of foregrounding, which is based on the idea of deviation from standard language norms, gaining significance in literary texts [28]. Such deviation manifests either qualitatively, through contextual incongruity, or quantitatively, through repetition. A pertinent example appears in *Hard Like Water* [29], where the protagonists engage in physical intimacy after emerging from a tomb. Describing this scene, Yan writes: “The revolution is the time and season of all miracles.” The metaphor adds a temporal and symbolic layer to the depiction of the revolution, illustrating how Yan’s writing invests revolutionary discourse with symbolic resonance beyond its immediate political context. Here, the characters’ emergence and bodily awakening function not merely as sensory or erotic descriptions, but as metaphors for rebirth and transformation. As Leech [28] argues, since literary style is intrinsically constituted by such “deviant” or “exceptional” features, identifying these anomalies is fundamental to literary analysis.

While foregrounding is often signaled by statistically significant linguistic deviations, the two are not synonymous [28]. Indeed, established foregrounding phenomena, such as alliteration, metaphor, simile, and syntactic irregularities, may not always exhibit statistical significance. Conversely, statistically salient features, including keywords and key semantic domains, do not inherently constitute foregrounding; their interpretive relevance requires qualitative assessment through close reading. Nevertheless, such deviations serve as vital heuristic signals that warrant investigation as potential indicators of foregrounding. In corpus analysis, deviation is defined as a linguistic feature demonstrating a statistically significant positive keyness score, occurring markedly more frequently in a target text than in a reference corpus [28]. To detect these deviations, Leech [28] employs Wmatrix [30], a computational tool designed to identify keywords and key semantic domains. Although Wmatrix quantifies statistical deviation rather than foregrounding itself, it provides the empirical data necessary to support deviation-based interpretations, thereby grounding the theoretical framework of foregrounding in quantitative evidence. The tool’s methodological versatility is further evidenced by its widespread application in diverse fields, ranging from comparative cultural studies [31] to character analysis [32]. In light of this, Leech’s framework [24,28], in conjunction with Wmatrix, offers a robust methodological foundation for examining linguistic deviation and foregrounding in Yan’s works.

## 3. Method of analysis

Corpus stylistics is defined as the application of stylistic theories and frameworks to the analysis of corpus data [26]. Fundamentally, the discipline bridges the descriptive observation of linguistic forms with interpretative analysis grounded in literary and linguistic theory, offering a systematic means to elucidate textual features and discourse-level meanings. Inspired by the methodological paradigms proposed by Leech [28] and Can and Cangır [27], this study adopts a corpus-based approach to examine the English translations of Yan Lianke’s novels, utilizing a reference corpus of contemporary Chinese fiction for comparative analysis. Since this study analyzes publicly available literary texts and involves no human participants, neither ethical approval nor informed consent was required.

This study addresses the following two research questions:

1) Which semantic domains in the English translations of Yan Lianke’s novels are statistically overused, and which of them can be identified as foregrounded based on combined quantitative and qualitative criteria?
2) How do the foregrounded semantic patterns, along with their associated lexical items, collocational behavior and contextual usage, function stylistically and contribute to the construction of *mythorealism* in the translated texts?

### 3.1 Corpora compilation

This study examines nine of Yan’s most famous and English-translated novels, including eight published novels and one novella. The decision to analyze English translations rather than the Chinese source texts is necessitated by technical constraints, specifically regarding the accuracy of automatic semantic tagging. While the English USAS tagger within Wmatrix is rigorously validated and robust [28], current semantic tagging resources for Chinese remain experimental. A recent evaluation by the USAS developers [14] reports that the standard rule-based Chinese tagger achieves a Top-1 accuracy of only 32.6%, hampered primarily by segmentation errors and limited lexicon coverage; even experimental neural models attain only 47.9%. Consequently, direct analysis of the Chinese source texts would introduce unacceptable levels of methodological noise. To extend the analytical affordances of semantic domain-based corpus stylistics to Chinese literature despite these deficits, this study employs English translations as a necessary methodological bridge. We acknowledge that this reliance on translation produces a “hybrid voice,” inextricably blending the author’s original style with the translator’s linguistic habits. However, we posit that a rigorously controlled semantic domain analysis can effectively retrieve the core thematic and stylistic dimensions of Yan’s work, which is underpinned by the following four strategies designed to minimize translator interference.

First, the study prioritizes reliance on stylistically faithful translations, predominantly those by Carlos Rojas, who is widely recognized for his “defamiliarizing” fidelity and stylistic sensitivity [33]. Second, the methodology employs semantic abstraction (or de-lexicalization) to mitigate the impact of specific lexical choices. Unlike keyword analysis, which is highly susceptible to a translator’s idiosyncratic preferences, semantic domain analysis operates at a higher conceptual level. Consolidating diverse lexical variants such as “belief,” “faith” and “conviction” into broader semantic fields (e.g., X2.1: Thought and belief), this study targets the text’s “aboutness” and conceptual density [28], which ensures resilience against minor translational shifts and captures the stable thematic structures underpinning the narrative.

Furthermore, a qualitative attribution filter is applied based on the distinct nature of the linguistic features under investigation. Drawing on Baker’s framework [34], a theoretical distinction is posited between the “forensic” linguistic habits of the translator and the “thematic” imperatives of the author. Baker argues that a translator’s style is primarily manifested in “subtle, unobtrusive linguistic habits which are largely beyond the conscious control,” such as average sentence length and type/token ratio. Therefore, this study excludes high-frequency grammatical domains (e.g., Z5: Grammatical bin, Z8: Pronouns) from the stylistic interpretation, attributing these syntactic markers to Rojas’s idiolect or general translational norms. In contrast, mythorealism is defined by its content, imagery, and semantic density, features encapsulated within content-heavy domains such as G1.2 (Politics) and S9 (Religion and the supernatural). While the translator exercises agency over specific lexical realizations, they lack the license to invent the recurrence of the imagery itself. Therefore, statistical saturation in these substantive semantic fields is interpreted as a distinct signature of Yan’s narrative world rather than a translational invention, allowing for the reconstruction of the author’s thematic and stylistic architecture to the greatest extent possible. Finally, a stylistically heterogeneous reference corpus is employed to neutralize individual translator idiolects, ensuring that statistical deviations isolate the author’s enduring stylistic signatures that have persisted through the translation process.

Nonetheless, this study acknowledges the potential influence of translator voice. In this regard, Section 4.2.1 briefly addresses the role of the translator, offering a preliminary discussion of how certain adjustments may be made without altering the core patterns and stylistic identity of the source text. The discussion offers insights from a semantic-structural perspective, providing a valuable lens for future stylistic and translation studies on translated literature. The specific details of the English translations selected for this study are provided in Table 1.

**Table 1:** Corpus of Yan Lianke’s English-translated Novels.

No	Translated Novels	Publication Year^a^	Translator	Type	Tokens
1	*Lenin’s Kisses*	2012	Carlos Rojas	Novel	169,779
2	*The Four Books*	2015	Carlos Rojas	Novel	111,497
3	*The Day the Sun Died*	2018	Carlos Rojas	Novel	114,344
4	*Heart Sutra*	2023	Carlos Rojas	Novel	107,110
5	*Dream of Ding Village*	2011	Cindy Carter	Novel	108,862
6	*The Years, Months, Days and Marrow: Two Novellas*	2015	Carlos Rojas	Novella	53,709
7	*Hard Like Water*	2021	Carlos Rojas	Novel	139,347
8	*The Explosion Chronicles*	2016	Carlos Rojas	Novel	144,351
9	*Serve the People!*	2007	Julia Lovell	Novel	40,642

^a^The publication years listed in the table refer to the release dates of the English translations.

On the other hand, to capture the semantic features of Yan Lianke’s translated works, this study established a comparative reference corpus comprising English translations of novels by 15 widely recognized contemporary Chinese authors. It includes 13 recipients of the Mao Dun Literature Prize, China’s most prestigious award for the novel, as well as winners of the Lu Xun and Lao She Literature Prizes. As these authors began their careers during the same period as Yan, they share comparable socio-cultural and historical backgrounds, ensuring that the reference corpus aligns both temporally and contextually with the texts under analysis, thereby enabling a valid synchronic comparison [28]. Given the prominence of regional writing and magical realism in Yan’s fiction [35], one representative full-length novel was selected from each author, prioritizing thematic and stylistic affinity. The rigorous selection process accounted for diversity in authorship and translation while controlling for publication era and genre. Accordingly, the reference corpus serves as a robust benchmark for assessing the specific semantic characteristics of the observed corpus. Details of the reference corpus are listed in Table 2.

**Table 2:** Contemporary Chinese Writers’ English Translated Novels Corpus.

No	Novel	Publication Year^a^	Translator	Author	Tokens
1	*A Dictionary of Maqiao: A Novel*	2003	Julia Lovell	Han Shaogong	145,943
2	*Decoded: A Novel*	2014	Olivia Milburn and Christopher Payne	Mai Jia	110,736
3	*Life*	2019	Chloe Estep	Lu Yao	70,420
4	*Nanjing 1937: A Love Story*	2002	Michael Berry	Ye Zhaoyan	140,910
5	*Peach Blossom Paradise*	2020	Canaan Morse	Ge Fei	123,397
6	*Red poppies*	2003	Howard Goldblatt and Sylvia Li-Chun Lin	Alai	147,962
7	*Red Sorghum*	2003	Howard Goldblatt	Mo Yan	132,578
8	*Ruined City: A Novel*	2016	Howard Goldblatt	Jia Pingwa	242,158
9	*Someone to Talk to*	2018	Howard Goldblatt and Sylvia Li-chun Lin	Liu Zhenyun	173,101
10	*The Ancient Ship: A Novel*	2008	Howard Goldblatt	Zhang Wei	166,428
11	*The Boat to Redemption*	2011	Howard Goldblatt	Su Tong	119,887
12	*The Last Quarter of the Moon*	2013	Bruce Humes	Chi Zijian	103,795
13	*The Song of Everlasting Sorrow: A Novel of Shanghai*	2008	Michael Berry and Susan Chan Egan	Wang Anyi	195,868
14	*Three Sisters*	2010	Howard Goldblatt and Sylvia Li-chun Lin	Bi Feiyu	101,700
15	*To Live: A Novel*	2003	Michael Berry	Yu Hua	69,352

^a^The publication years listed in the table refer to the release dates of the English translations.

### 3.2 Semantic pattern identification via Wmatrix and LancsBox

To determine whether a semantic domain exhibits foregrounding involved a two-step process combining quantitative identification with qualitative evaluation. First, Wmatrix [30] was used for automatic semantic tagging to identify linguistic deviations, specifically semantic domains that are statistically overused in the Yan corpus compared to the reference corpus. The concept of key semantic domains is derived from “keyword analysis” [36], which refers to domains that occur more frequently in one corpus than in another. When comparing two corpora, they reveal relatively more prominent semantic themes rather than isolated lexical items. The analysis can delineate the shared semantic functions underlying synonymous variants, making it particularly suitable for identifying underlying discourse topics or conceptual patterns. Compared to word frequency analysis, it offers deeper insights into the semantic characteristics of a given text or authorial style and better reflects stylistic tendencies.

Upon upload to Wmatrix, the sub-corpora were subjected to automatic annotation, assigning each lexical unit a part-of-speech (POS) tag and a semantic domain tag (USAS). For instance, “Yan Lianke” was tagged as a singular proper noun and categorized within the semantic field of personal names. Wmatrix then performed a keyness analysis to identify semantic deviations by comparing relative frequencies between the target and reference corpora, which calculated both Log-Likelihood (LL) and Log Ratio scores, ranking domains by effect size to reveal patterns of overuse and underuse. As a measure of effect size, the Log Ratio is particularly advantageous for corpus comparison; it not only quantifies the magnitude of frequency differences but also provides an objective criterion for narrowing the dataset for subsequent qualitative analysis [37].

McIntyre and Walker [26] typically require an LL value above 15.13 and a log ratio of at least 1 for overused domains. While adhering to the former, this study adopts Leech’s [28] framework to refine the latter, treating computational output as a “heuristic strategy,” a set of “good bets” for investigation, rather than a rigid mandate. Given the inherent 8% tagging error rate in the system, a strict 1.0 cut-off risks arbitrarily severing vital components from their interconnected semantic domains. The danger is empirically evident in the data. For instance, while the domain *Politics* (G1.2) is statistically salient (Log Ratio 1.77), its semantic correlate, *Government* (G1.1), falls marginally below the standard threshold (Log Ratio 0.92). A rigid exclusion of the latter would capture the general political discourse while stripping away its institutional framework, resulting in a fragmented representation of the political narrative. Hence, adjusting the threshold to 0.9 serves as a pragmatic measure to retain these “logical companions,” recovering seven marginal foregrounded semantic domains, thereby ensuring the full coherence of semantic patterns.

In addition to the semantic domain analysis, this study also investigated the key lexical items within each domain (limited to the top ten), reporting both their raw frequencies and relative frequencies (expressed as percentages of the total word count). These items were subsequently subjected to qualitative scrutiny using the GraphColl function in LancsBox [38], which identifies and visualizes their collocational networks and co-occurrence patterns. Through close reading and contextual analysis, the study examined the semantic and stylistic functions of these items in the translated texts, offering deeper insights into their discursive significance and their contribution to the construction of style.

The GraphColl tool in LancsBox was used to identify and visualize collocations in both tabular and network formats, facilitating the analysis of collocates for key lexical items within specific semantic domains. By examining the collocational patterns surrounding these target items, the tool provides essential contextual data that supports more precise interpretive judgments. To ensure methodological rigor, the analysis adopted parameters recommended by Brezina et al. [39], employing the Mutual Information (MI) score as the primary statistical measure. MI uses a logarithmic scale to quantify the ratio of observed collocation frequency to expected random co-occurrence, indicating the strength of lexical association [40]. As a normalized measure, MI facilitates cross-corpus comparisons by prioritizing infrequent but strongly associated word pairs while minimizing the influence of high-frequency function words, such as articles and auxiliaries [27]. Given the study’s objective to detect distinct, high-strength lexical combinations, the integration of MI scores was methodologically justified. Accordingly, parameters were configured to retrieve the top 10–30 collocations (contingent on statistical relevance) based on raw forms, applying a frequency cut-off of 3 and a span of 3-/ + words from the node. The analysis focused on the top three key lexical items within each target semantic domain (with a minimum frequency of 150), examining their MI-ranked collocates and associated concordance lines to support qualitative interpretation. These relationships are visualized as GraphColl networks (see figures), where the central node represents the target item and the surrounding nodes represent collocates; radial proximity indicates the strength of association, with shorter distances reflecting higher MI values and stronger lexical ties.

Wmatrix USAS tagging achieves roughly 92% accuracy, implying an inherent margin of error due to polysemy and context dependence [28]. For example, *liberal* is sometimes misclassified as *Allowed* (S7.4+) instead of *Politics* (G1.2) [30]. To address this, this study adopts a three-stage protocol: quantitative identification, manual verification, and error pruning. By supplementing statistical screening with qualitative analysis (concordance reading and GraphColl MI), we identify and remove algorithmic artifacts. As shown in Section 4.1 with the exclusion of the *Degree* (A13) domain, the verification ensures that the final foregrounded patterns reflect genuine semantic coherence and stylistic interpretability. Following these procedures and the exclusion of misclassified domains, a semantic domain is identified as foregrounded if it satisfies the criteria: 1) statistical salience (i.e., significant frequency deviation); 2) thematic relevance (e.g., pertaining to history, nature, or psychological experience); and 3) stylistic distinctiveness (e.g., referential ambiguity, symbolic resonance, or cultural embeddedness). Then, qualifying domains are synthesized into broader semantic patterns based on their conceptual affinities and stylistic functions. These patterns constitute the empirical foundation for identifying and interpreting the stylistic manifestations of mythorealism in Yan’s translated fiction.

## 4. Results and analysis

As explained in Section 3.2, the output reports observed raw frequencies (Freq. (T) for the Corpus of Yan Lianke’s English-translated Novels and Freq. (R) for the Contemporary Chinese Writers’ English Translated Novels Corpus), relative frequencies (Rel. freq. (%)) and statistical significance via log-likelihood (LL). Table 3 presents a ranked list of overused semantic domains in the target corpus based on log ratio values (effect size). It should be noted that the Grammatical Bin domain (Z5) was excluded from the stylistic analysis, as it encompasses high-frequency functional items that, consistent with Baker’s framework [34], are attributed to the translator’s linguistic habits rather than the author’s thematic architecture.

**Table 3:** Overused semantic domains in the target corpus relative to the reference corpus.

Item	Freq. (T)	Rel. freq. (%)	Freq. (R)	Rel. freq. (%)	LL	LogRatio	Semantic Domain
G1.2	1457	0.15	886	0.05+	860.31	1.77	Politics
T3---	725	0.08	487	0.02+	377.58	1.62	Time: New and young
A13	1283	0.14	981	0.05+	554.02	1.44	Degree
H1	3032	0.32	3635	0.19+	480.43	1.19	Architecture, houses and buildings
M7	8992	0.95	8202	0.42+	2842.15	1.18	Places
F4	2173	0.23	1996	0.10+	677.25	1.17	Farming and Horticulture
O4.3	5665	0.6	7763	0.40+	543.45	1.01	Color and color patterns
W2	1918	0.2	2026	0.10+	435.97	0.98	Light
H2	6121	0.65	7480	0.38+	910.89	0.96	Parts of buildings
L1-	3224	0.34	3968	0.20+	468.36	0.95	Dead
S9	3250	0.34	4306	0.22+	359.36	0.94	Religion and the supernatural
G1.1	2009	0.21	2359	0.12+	339.87	0.92	Government
W1	2106	0.22	2495	0.13+	346.56	0.91	The universe
L3	4728	0.5	5242	0.27+	945.64	0.90	Plants

### 4.1 From statistical identification to semantic pattern construction

While the identification of overused semantic domains in the target corpus, those exhibiting statistically significant deviations, can serve as a starting point for detecting stylistic foregrounding, it is important to note that not all semantic deviation necessarily equates to foregrounding. Thus, it is necessary to first eliminate overused domains that lack foregrounding potential.

A qualitative examination of the semantic domains listed in Table 3 indicates that *Degree* (A13) and *Time: New and Young* (T3) do not constitute foregrounded features. Regarding the *Degree* domain, Wmatrix identifies *as* as the sole significant lexical contributor. Further analysis via LancsBox reveals that of the 7,411 instances of *as*, approximately 32% (2,348) appear in the “as…as” construction. While this structure facilitates similes and rhetorical intensification, valid stylistic features, the word *as* is highly polysemous. In many instances, it functions as a temporal, causal, or conditional conjunction, making its classification under *Degree* erroneous. This conflation is likely a byproduct of the tagger’s approximate 92% accuracy rate [28]. A similar discrepancy affects the *Time: New and Young* domain, which is driven primarily by the item *revolutionary*. Collocational analysis demonstrates that its strongest associations are with *successor* (MI = 10.8), *comradely* (MI = 10.7), and *politician* (MI = 10.5). Contextual scrutiny confirms that *revolutionary* functions here as a component of political discourse rather than as a temporal marker. As a result, both domains lack the requisite semantic coherence and salience to be considered foregrounded.

Following the exclusion of semantic domains that lack foregrounding features, this study groups together thematically related and foregrounded domains into broader semantic patterns. Through qualitative analysis, five such patterns are identified: political discourse, spatial narrative, natural environment, color symbolism and the supernatural. Political discourse emerges as a dominant pattern, encompassing key semantic domains such as *Politics* (G1.2) and *Government* (G1.1). Spatial narrative draws on domains including *Parts of Buildings* (H2) and *Architecture, Houses and Buildings* (H1), covering a range of spatial expressions that help readers construct and interpret both physical and symbolic spaces within the narrative world. The natural environment pattern incorporates domains such as *Location* (M7), including items like *village*, *county*, *town*, *villagers* and *city*, along with *Plants* (L3) and *Farming and Horticulture* (F4). These domains are closely interrelated and together provide the environmental backdrop for Yan’s storytelling, reflecting his emphasis on regional settings. Color symbolism is primarily represented by the domain of *Color and Color Patterns* (O4.3), while the supernatural pattern includes domains such as *Religion and the Supernatural* (S9) and *The Universe* (W1), contributing to the surreal dimensions of his work.

The five semantic patterns identified above highlight distinct foregrounded stylistic features in Yan Lianke’s novels, characterized by their rural and political settings as well as a surreal portrayal of China’s societal and cultural transformations. However, given that the natural environment pattern functions primarily as a narrative backdrop and has already garnered significant scholarly attention [4,8,41], this study prioritizes the remaining four patterns for subsequent qualitative analysis. Notably, statistically salient domains such as *Dead* (L1-) and *Light* (W2) are excluded from the primary patterns. Despite sharing log ratios below 1.0 with retained instances like *Government* (G1.1), they are omitted because they lack the “logical companions” needed to constitute robust semantic patterns. While these domains reflect stylistic dimensions warranting dedicated study, such as the death-centered narratives [42], they are deferred to future research to ensure the study remains focused on the semantic patterns.

### 4.2 Qualitative analysis of selected semantic patterns

This section investigates the stylistic features and thematic functions of the foregrounded semantic patterns, focusing specifically on political discourse, spatial narrative, color symbolism, and the supernatural. To ensure analytical depth within spatial constraints, detailed analysis is restricted to the single semantic domain exhibiting the highest log ratio within each pattern, which serves as a representative exemplar of the broader stylistic trend. For each selected domain, the ten most frequent lexical items are scrutinized regarding their collocational networks and contextual usage. Moreover, Section 4.2.1 presents a supplementary case study within the political discourse pattern to address the challenge of translator “fingerprints” [34] noted in Section 3.1. In the absence of reliable semantic tagging tools for Chinese, the analysis precludes direct domain-level comparisons between source and target texts. Instead, adopting Mastropierro’s [43] framework for cross-linguistic stylistic comparison, the study focuses on the collocational behavior and contextual functions of specific lexical items, which enables a precise assessment of stylistic transformation and thematic continuity within the parallel corpus.

#### 4.2.1 Political discourse.

Wmatrix identifies *Politics* (G1.2) and *Government* (G1.1) as prominent semantic domains, underscoring the corpus’s central preoccupation with political themes. The statistical salience aligns with Yan’s designation as a “banned author” in China [2], a status stemming from his persistent interrogation of political ideologies and sensitive historical events. His narratives frequently foreground epochs often marginalized in official discourse, such as the Cultural Revolution and the Great Leap Forward. As a result, the political dimension of mythorealism has become a primary focus of Western scholarship [17]. To further investigate this thematic strand, the following analysis isolates the *Politics* (G1.2) domain to examine how the texts construct and critique ideological discourse.

**Fig 1:**
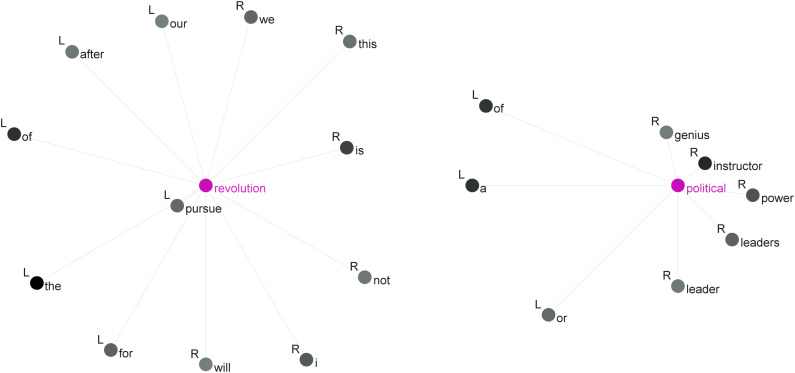
Collocations of *revolution* and *political.*

Table 4 presents the top ten lexical items within the heavily utilized semantic domain of *Politics*. Notably, the term *revolution* occurs with such frequency that Wmatrix consolidates noun phrases like *the revolution* and *of revolution* into a single lexical item. In this context, *revolution* primarily refers to the Great Leap Forward, a campaign initiated in China from 1958 to 1960 to boost industrial and agricultural production, as well as the Cultural Revolution*,* a radical political movement in the 1960s marked by internal social upheaval. Other lexical items in the table, such as *vote*, *socialist*, and *feudal*, serve to characterize the concepts of *revolution* and *political*. To further explore the stylistic features of these two key terms, their collocational networks are analyzed. Fig 1 below visualizes the statistically significant collocates of the node words *revolution* and *political*.

**Table 4:** *Politics* in pattern of political discourse.

Item	Tag	Freq.	Rel. freq.
The revolution	G1.2	300	0.03
Political	G1.2	183	0.02
Of revolution	G1.2	47	0.00
Vote	G1.2	47	0.00
Socialist	G1.2	47	0.00
Revolution	G1.2	37	0.00
Feudal	G1.2	33	0.00
Politics	G1.2	31	0.00
Votes	G1.2	31	0.00
Socialism	G1.2	30	0.00

As illustrated in Fig 1, the strongest collocate of the node word *revolution* is *pursue* (MI = 10.3), followed by *is* (MI = 5.6) and *our* (MI = 4.5). In the outer collocational layer, several prepositions—such as *after*, *for* and *of*—serve to specify particular aspects or attributes of *revolution*, often offering descriptive elaborations or contextual definitions. On the other hand, lexical items most strongly associated with the node word *political* include *instructor* (MI = 12.0), *power* (MI = 9.9) and *leaders* (MI = 9.8). These political*-*related terms frequently function as plot drivers or carry metaphorical and literary connotations. To gain a more precise understanding of these collocational patterns, we further examine their concordances, focusing on recurrent phrases such as *pursue revolution* (out of 33 instances), *revolution is* (out of 86 instances) and *our revolution* (out of 27 instances).

(1) “Company leader told everyone to seize their spirit in order to search for flaws and to imprison their thought in order to pursue revolution” [29].
(2) “He said, ‘Pursue revolution and protect production, because if production is insufficient, how can you pursue revolution!’” [29].
(3) “Revolution is a foundation, and love is a house built upon that foundation” [29].
(4) “The revolution is the source and the engine of all miracles” [29].
(5) “Love grants our revolution endless strength, and makes our will stronger” [29].

As the concordance data illustrates, the concept of revolution permeates the characters’ existence, functioning as a central force that foregrounds this semantic domain. Of the 86 instances of the syntactic structure “revolution is,” 46 (approximately 53%) employ metaphorical constructions. These metaphors, which conceptualize revolution variously as a *stem*, *ship*, *ocean*, *field*, *harvest*, and *fishnet*, collectively underscore its essential yet demanding nature. Specifically, organic metaphors like *stem* and *harvest* frame revolution as a life-sustaining force and a source of reward, emphasizing its vitality in national development. Meanwhile, images of the *ship*, *ocean*, and *fishnet* evoke vastness and coordination, suggesting that revolution necessitates persistence, sacrifice, and communal labor. Through the figurative language, *revolution is* rendered as both indispensable and arduous, exacting total commitment to achieve collective ends. Furthermore, the recurrence of the phrase *our revolution* signals a profound sense of collective identity and emotional investment. Characters frequently internalize the revolution as a source of “warmth,” “love,” “will” and “strength,” illustrating the deep penetration of political ideology into personal experience. However, while these expressions ostensibly project unified enthusiasm, the broader narrative, exemplified by the opening excerpt, invites critical reflection on the ultimate human cost of such revolutionary zeal. Hence, a deep understanding of the political backdrop is indispensable for interpreting the stylistic features of Yan’s novels and the historical evolution they critique.

While this study primarily focuses on the semantic patterns identified in Yan’s works, it is necessary to briefly address translators’ fingerprints in shaping the stylistic presentation and mediating certain narrative effects. To this end, a preliminary collocational analysis (MI > 3, within a span of 5-/ + words) was conducted on the keyword *revolution* (Chinese: *geming* [革命]) within the foregrounded pattern of political discourse, with Carlos Rojas’s English translation of *Hard Like Water* as a case study. The analysis reveals several highly similar collocational patterns in both the Chinese text and the English translation, for instance, *zhuageming* [抓革命, pursue revolution] and *cushengchan* [促生产, promote production], among the top ten collocates of the keyword. These collocates not only reflect the collectivist and mobilizing function of revolution as a driving force behind social action and productivity but also consistently rank among the top three in terms of mutual information scores. Such overlap suggests a notable degree of conceptual stability across languages, indicating that the translator has largely preserved the semantic role of revolution.

While the core collocational network remains stable, subtle variations in statistical prominence are observable in peripheral associations. In the original Chinese, *revolution* frequently co-occurs with highly embodied and emotionally charged terms such as *toulu* [头颅, skull], *zheng* [症, symptom] and *ai* [爱, love], creating a satirical narrative mode centered on individual experience, bodily sacrifice, and mundane social interactions. In the English translation, these embodied elements are not erased but rather modulated in their statistical weight. While present, they exhibit fluctuating MI scores compared to the source text. More abstract and institutional terms, such as *proletarian*, *law*, and *cultural*, exhibit amplified statistical salience in the English collocation list, with MI scores roughly twice as high as those in their Chinese counterparts. It indicates not a fundamental divergence, but a shift in stylistic intensity, suggesting that the translator positions *revolution* within a broader ideological and structural discourse while retaining its underlying semantic fabric. The contrast highlights a stylistic strategy of reweighting: while preserving the core semantic structure of revolution, the translator adjusts the prominence of specific associations, slightly downplaying the satirical intensity of the bodily narrative to accentuate institutional legitimacy and historical rationality. Such adjustments render the translation more accessible to Anglophone readers in terms of cultural expectations.

To enhance the transparency and credibility of the findings, additional bilingual concordance samples containing the keyword “revolution” from *Hard Like Water* have been included in the Supplementary Information (S1 File). These examples further demonstrate that the identified semantic patterns primarily reflect Yan’s distinctive stylistic and thematic features persisting through translation. Moreover, this case demonstrates that even in translated texts, corpus-based stylistic analysis remains a valid and productive method for identifying the stylistic matrix of Yan’s work, offering a quantifiable pathway for tracing translator’s fingerprint and stylistic modulation across languages in future studies.

#### 4.2.2 Spatial narrative.

The spatial narrative pattern encompasses two semantic domains (H2, H1), which primarily cover the settings of character activities and events, as well as related entities, in Yan’s novels. This section focuses on *Parts of buildings*, which more typically represents spatial narrative properties and ranks second by log likelihood within this pattern, for discussion.

**Fig 2:**
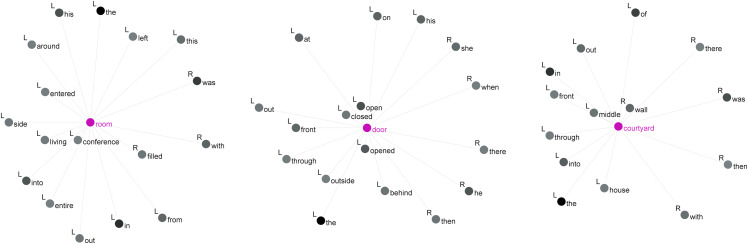
Collocations of *room*, *door* and *courtyard.*

Table 5 presents the top ten key items associated with the key semantic domain of *Parts of buildings*, primarily concerning various architectural imagery. The frequency values of these items are relatively high when compared with the other key items from the other key semantic fields, ranging from 0.09 to 0.02. A deeper analysis of the contextual environments of the three highest-frequency items is conducted by examining their collocational networks to identify stylistic elements. The accompanying graphs illustrate the direction of collocates (i.e., left, right, or middle position) for the keywords *room*, *door* and *courtyard*. Additionally, the frequency of each collocate is represented by the intensity of its color and the strength of the associations is indicated by the distance of the connecting lines (Fig 2).

**Table 5:** *Parts of buildings* in spatial narrative pattern.

Item	Tag	Freq.	Rel. freq.
Room	H2	858	0.09
Door	H2	775	0.08
Courtyard	H2	696	0.07
Wall	H2	473	0.05
Window	H2	370	0.04
Hall	H2	358	0.04
Gate	H2	304	0.03
Walls	H2	238	0.03
Floor	H2	204	0.02
Doorway	H2	202	0.02

For *room*, the strongest collocations are the noun adjuncts *conference* (MI = 8.8) and *living* (MI = 7.2), followed by words strongly associated with it, such as *entered*, *filled*, *side*, *into*, *left* and *around*. Regarding the second target item, *door*, the most potent collocates are the verbs *open(ed)* (MI = 8.2) and *closed* (MI = 8.0), along with spatial prepositions like *behind* (MI = 5.9), *outside* (MI = 5.9), *front* (MI = 5.6), *through* (MI = 4.8) and *out* (MI = 3.6). Thirdly, words associated with *courtyard* include *wall* (MI = 7.0), *middle* (MI = 6.7), *gate* (MI = 6.6), followed by spatial prepositions such as *into*, *through*, *front*, *out* and *in*. The statistical prominence of directional prepositions signals a narrative preoccupation with positionality and movement. Qualitatively, these terms serve to demarcate physical thresholds, a linguistic pattern concretized through architectural symbols that imbue the dichotomy of “inside” and “outside” with multilayered significance, spanning the political, social, and existential spheres.

Representative concordance lines for the clusters *conference room* (out of 26 instances) and *living room* (out of 32 instances) are as follows:

(1) “We stood in the conference room against a wall positioned between the door and the window and amid the sound of militiaman’s footsteps silently held our breath as we did that thing” [29].
(2) “We would first go to the conference room and... we would silently kiss...” [29].
(3) “Several people from the city government stayed behind in the conference room... the room was full of the smell of baijiu” [44].
(4) “Eventually, the living room of the Zhu family’s three-story house would be transformed into a gallery” [44].

As indicated by these clusters, a marked spatial separation is constructed. The *conference room*, originally designed for discussing political matters and conducting official business, becomes the setting for the protagonist’s affair in *Serve the People!*. It is the site of the forbidden love affair between *Liu Lian*, the young and attractive wife of a powerful Division Commander in Communist China and *Wu Dawang*, her household’s lowly servant. It also serves as a place where government officials drink. In the novel *Dream of Ding Village*, the protagonist’s family living room becomes a gallery after their endeavors in selling blood and amassing wealth. Regardless of whether it’s a conference room or a living room, the spaces inside and outside the room represent different social scenes, each physical space embodying distinct characters or events. As architectural symbols in spatial narratives, the imagery of the *room* bears significant stylistic meaning. The rigid division of physical space into interior and exterior does more than define geography; it highlights the communication barriers between these two worlds, ultimately mirroring the profound divides between psychological and social spaces.

#### 4.2.3 Color symbolism.

As in previous sections, this part presents both the raw and relative frequencies of lexical items within the *Color and color patterns* domain. It then illustrates the networks of frequently co-occurring items and provides representative concordance lines, offering insights into their stylistic functions and contextual usage.

**Fig 3:**
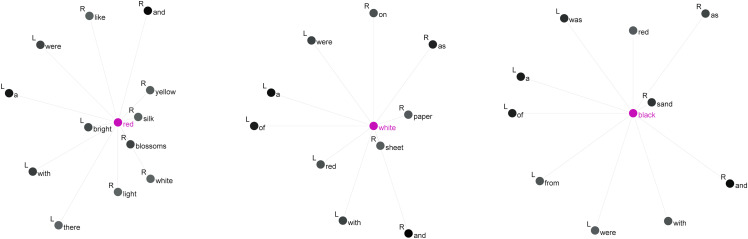
Collocations of *red*, *white* and *black.*

Table 6 presents the ten most frequent lexical items within the *Color and color patterns* semantic domain. The relative frequencies of these items are notably high compared to those in other key domains, ranging from 0.13% to 0.01%. Among them, *red* (0.13%) stands out with a marked lead, appearing nearly twice as often as *white* (0.06%) and *black* (0.05%), which rank second and third, respectively. In light of the distribution, the collocational networks of *red*, *white*, and *black* are further examined to explore their stylistic significance. Fig 3 below visualizes the statistically significant collocations associated with these node words.

**Table 6:** *Color and color patterns* in color symbolism pattern.

Item	Tag	Freq.	Rel. freq.
Red	O4.3	1187	0.13
White	O4.3	590	0.06
Black	O4.3	507	0.05
Yellow	O4.3	413	0.04
Bright	O4.3	387	0.04
Green	O4.3	316	0.03
Pale	O4.3	220	0.02
Blue	O4.3	128	0.01
Gray	O4.3	126	0.01
Golden	O4.3	111	0.01

As shown in Fig 3, the item *red* is strongly associated with collocates such as *silk* (MI = 7.8), *blossoms* (MI = 7.7), and *bright* (MI = 7.4), as well as other color terms like *yellow* (MI = 6.7) and *white* (MI = 5.7). Secondly, the node word *white* is strongly collocated with terms like *sheet* (MI = 7.9), *paper* (MI = 7.1), and *red* (MI = 5.7). The first two are nouns modified by *white*, while the last is a color term co-occurring with *white*. Finally, for the node word *black*, the strong collocates are *sand* (MI = 9.3) and *red* (MI = 5.6), with the former being a noun modified by *black* and the latter a co-occurring color term. It is notable that both *white* and *black*, as some of the earliest evolved basic color terms [45], appear in Yan’s novels in conjunction with the basic color term *red* and have strong collocational relationships with it. Hence, we continued our examination of the item *red*, including concordance lines for the clusters *red silk* (out of 48 instances), *red blossoms* (out of 105 instances), and *bright red* (out of 84 instances).

(1) “Before he died, Zhao Dequan had simply wanted to give his wife the red silk jacket he had promised her as wedding gift” [46].
(2) “The Child grabbed five more red blossoms and asked, ‘Has anyone else made up the mind?’” [47].
(3) “Just as the sun was coming up, the Child has spread the red blossoms over the ground and then nailed himself to the cross” [47].
(4) “First there was a bright red song, followed by countless cries and screams, as though from a mental asylum...” [29].
(5) “I saw that the bright red excitement on her face showed no sign of diminishing...” [29].

Through these concordance lines, we can intuitively perceive Yan’s preference for the color red in his novels, which is closely associated with women and cultural events. The first example is from *Dream of Ding Village*, where Ding Liang, suffering from AIDS and nearing death, wishes to give his wife Lingling a *red silk* jacket, a wedding gift he had promised at their marriage. The *red silk* jacket, vibrant and beautiful, symbolizes the couple’s fond memories and hopes for future life. However, as Ding Liang faces death, the simple wish becomes a poignant reminder of the vast gap between hope and reality, with all regrets encapsulated in this *red silk* jacket.

Drawing from *The Four Books*, the subsequent examples illustrate how the motif of *red blossoms* is inextricably linked to the figure of *The Child*. As the authoritarian warden of the re-education camp, *The Child* utilizes these tokens to enforce compliance, distributing them not only for obedience but also as rewards for reporting the transgressions of fellow inmates. Because the blossoms grant temporary privileges and the ultimate promise of freedom, they become objects of intense obsession. The desire gradually transforms the prisoners into instruments of surveillance; in their pursuit of recognition, they turn against one another. Thus, the intellectuals, though victims of confinement, are rendered complicit as collaborators within the system. The narrative culminates in a scene of tragic irony: *The Child* releases the prisoners and scatters the blossoms before crucifying himself, an act serving as both a paradoxical liberation and a final reinforcement of the novel’s grim absurdity. Culturally, *red blossoms* are traditional symbols of praise and encouragement bestowed upon children. Yan subverts this innocent imagery with deep irony. Under *The Child’s* regime, they are transmuted from tokens of approval into mechanisms of discipline, surveillance, and moral compromise. By distorting this cultural emblem, Yan employs the blossoms as a vehicle for social critique, revealing how systems of reward restructure human relations and corrode solidarity. In this sense, the red blossoms function simultaneously on literal, cultural, and allegorical levels, dramatizing the interplay of individual desire and institutional power while anchoring Yan’s broader critique of authoritarian structures and human frailty.

In addition, an examination of 84 instances of the phrase *bright red* reveals its semantic versatility. Beyond its literal function, qualitative verification identifies that *red* operates metaphorically in 18 cases (approximately 21%), modifying nouns from the domains of sound, emotion and abstraction, such as *melodies*, *songs, revolution*, *splendor*, and *scent*. The statistical anomaly, characterized by the collocation of visual adjectives with auditory or abstract nouns, points to a distinct stylistic strategy. Through close reading, these collocational patterns are identified as synesthesia, epitomized by the fourth and fifth examples: the *bright red song* and *bright red excitement* from *Hard Like Water*. Previously, the characters’ erotic interactions were confined to a private, corporeal sphere, exemplified by the obsession with *red toenails*. The intrusion of the *bright red song*, however, shatters that isolation, signaling the forcible colonization of individual sensory experience by the grand narrative of revolutionary discourse. Here, *bright red* is not merely a symbol of revolutionary fervor; it acts as a catalyst inciting madder, bolder nakedness, thereby orchestrating an absurd confluence of private eroticism and public fanaticism. Crucially, the *countless cries and screams* that follow unveil a darker dimension of red, one of despair and destruction. Upon returning to the county seat, the protagonist finds a world “turned upside down,” marked by *bright red drops* and the *stench of blood*. This sharp transition from “song” to “scream” dramatizes the complex symbolic spectrum of red in Chinese socio-cultural narratives, where it simultaneously signifies soaring ideals and the crushing weight of reality. By employing this synesthetic technique to visualize the auditory and corporealize the political, Yan exposes a tragic symbiosis within the historical context: euphoric political melodies do not merely mask social violence but actively precipitate it.

#### 4.2.4 The supernatural.

Yan Lianke’s literary method is referred to as “mythorealism” where “real” denotes reality and “-ism” describes a system of theory, school, belief, or behavior [11]. “Mytho” signifies “God” or “Immortal,” representing supernatural powers or life. Although Yan Lianke’s mythorealism may predominantly be manifested in his narrative style and perspective, such as in *Dream of Ding Village*, which is narrated from the viewpoint of a deceased young boy, this study attempts to explore the characteristics of Yan’s mythorealism through semantic resources and stylistic features. As seen in Table 3, the supernatural pattern includes two foregrounded semantic domains (S9, W1). The following sections continue to investigate these, using the domain of *Religion and supernatural* as an example, to examine its lexical items and concordance lines.

**Fig 4:**
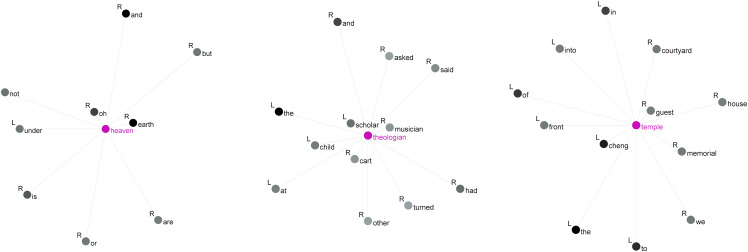
Collocations of *heaven*, *theologian* and *temple.*

In the semantic domain of Religion and Supernatural, there are a total of 208 lexical items, and Table 7 displays the ten items with relatively high frequency. These words convey the breadth and diversity of the religious and supernatural realms, covering aspects ranging from practitioners in Eastern and Western religions to objects of faith. Each lexical item is associated with a specific religious tradition or belief system, collectively highlighting how religious and supernatural phenomena are foregrounded in Yan’s novels. Next, we will explore the strong collocational relationships of the top three ranked items, *heaven* (0.02), *theologian* (0.02) and *temple* (0.02), as shown in Fig 4.

**Table 7:** *Religion and supernatural* in the supernatural pattern.

Item	Tag	Freq.	Rel. freq.
Heaven	S9	205	0.02
Theologian	S9	185	0.02
Temple	S9	177	0.02
Spirit	S9	154	0.02
Deities	S9	152	0.02
Religion	S9	143	0.02
Religious	S9	129	0.01
Buddhist	S9	117	0.01
God	S9	109	0.01
Pastor	S9	100	0.01

Here, we can see that the node word *heaven* is strongly collocated with the interjection *oh* (MI = 11.0), the contrasting term *earth* (MI = 10.4) and the preposition *under* (MI = 6.1). Secondly, the node word *theologian* is strongly associated with *Scholar* (MI = 8.1), *Musician* (MI = 8.0), *cart* (MI = 7.8) and *Child* (MI = 6.4), all of which are characters from the book *The Four Books*. The third node word, *temple*, has strong collocations with words like *guest* (MI = 10.0), *Cheng* (MI = 8.9) and *memorial* (MI = 7.9), primarily related to temple activities, and surrounded by prepositions such as *in*, *into*, *of*, *front*, and *to*, creating a new physical space. In the following section, we will continue to explore the concordance lines using the top three collocates of the most frequently used node word, *heaven* (out of 141 instances).

(1) “The world is so still. Everything under heaven is so still....” [48].
(2) “It was as though he finally felt safe now that all matters under heaven were in harmony and peace” [48].
(3) “Heaven, oh heaven! Earth, oh earth! The purple clouds and angel-shaped white clouds hovered in place directly above the Child’s head” [47].
(4) “And therefore felt that God, Heaven, and the Holy Spirit were going in opposite directions” [47].

“Heaven” is a typical religious term; in most religions, heaven is viewed as a transcendent, idealized place, representing supreme goodness and eternal peace. In Yan’s novels, heaven often appears alongside imagery such as *harmony* and *peace*, representing the psychological world and state of the characters, an idealized state of being that often conflicts with reality. As the first and second examples illustrate, the terrestrial world relies on the sanctity of heaven for inner peace. Yet, the very yearning underscores the cruelty of the characters’ lived experience. The third and fourth examples, drawn from *The Four Books*, center on the novel’s conclusion, where the narrative explicitly likens The Child’s self-sacrifice to that of Jesus (“Like Jesus, the Child nailed himself to a cross”). The accompanying exclamation “Heaven, oh heaven!,” expresses awe at this tragic spectacle while suggesting a redemptive quality, both political and religious, that transcends secular existence. The sanctity is visually reinforced by purple and white *angel-shaped clouds* that mirror the celestial domain. Furthermore, given that *heaven* signifies divine will or providence in Chinese culture, The Child’s shift from wielding absolute secular authority to suffering a religious death serves as a pivotal narrative device. Through the transition, Yan exposes the profound absurdity of the reality he depicts, merging political critique with metaphysical transcendence.

The fourth example, derived from the “New Myth of Sisyphus” manuscript within *The Four Books*, offers a philosophical reimagining of the Greek myth imbued with mythorealist color. Unlike the original punishment where Sisyphus pushes a boulder up a mountain, God alters the rules in this variant: Sisyphus must push the boulder down. The inversion compels Sisyphus to fix his gaze on the rough soil, turning his back to the sky. Consequently, the harder he labors, the further he becomes, physically and psychologically, estranged from *Heaven* and *Holy Spirit* located above, which serves as a poignant allegory for the fate of intellectuals in the Re-Education District. Here, “Heaven” symbolizes truth, ideals, and redemption. The forced labor is not only futile but contrary to natural laws, mirroring the downward push. Such absurd toil inflicts more than physical torment; by forcing intellectuals to face the earth in endless, head-bowed labor, the system severs their connection to the stars, leaving them feeling utterly forsaken by the spiritual truth they once held dear. In summary, religious and supernatural elements play a pivotal role in Yan Lianke’s novels, often serving as essential vehicles for fully understanding the symbolic significance and inner worlds in his storytelling.

## 5. Discussion

Yan Lianke [49] observes that “the mundane aspects of the secular world are the fertile ground where the spiritual elements and reality in literature grow and flourish.” However, the theoretical ambition of mythorealism extends far beyond mere observation. At its core, it seeks to transcend traditional realism, which relies on the “surface causal logic of everyday life,” by employing “inner causality” to excavate the “invisible reality” concealed beneath appearances [11]. A close analysis of Yan’s novels demonstrates that he consciously orchestrates five foregrounded stylistic patterns across multiple semantic domains, integrating them into the very fabric of his literary practice.

Political discourse (G1.2, G1.1) occupies a pivotal position in Yan’s oeuvre, a prominence Xie [3] attributes to the formative influence of the political upheavals witnessed during the author’s youth. In service of the “invisible truth,” such discourse offers restrained yet incisive commentary on historical traumas such as the Cultural Revolution and the Great Leap Forward. Rather than serving merely as a historical backdrop, it functions as a symbolic network elucidating the “truth obscured by representation.” Through revolutionary metaphors, collective rhetoric, and ritualized expression, Yan transmutes individual passions, sacrifices, and desires into a shared spiritual experience. Recurrent metaphorical constructions, notably the syntactic formula *revolution is*, shift the narrative focus from linear political causality to the revelation of spiritual and ethical truths. Thus, characters navigating the rhetorical force bear the weight of lived reality while engaging in a profound quest for meaning and redemption. The dynamic underscores mythorealism’s dual orientation toward both the historical and the spiritual. As Song [19] and Niu [41] observe, the shared revolutionary fervor among the populace reflects a form of collective unconscious. By foregrounding the “red revolutionary language,” Yan exposes how the human psyche is reshaped and distorted by historical contexts, articulating a critique that penetrates official narratives to reveal the underlying truth of the human condition.

In Yan’s narratives, the natural environment (M7, L3, F4) features prominently, providing a dynamic backdrop for his characters’ lives. Frequent depictions of villages and rural landscapes not only establish a tangible reality but also anchor the surreal elements of his fiction in a recognizable setting. This pattern reflects his profound attachment to the countryside; indeed, virtually all his extreme narratives engage with over half a century of China’s modern revolutionary history and rural social transformation [50]. Here, rural culture acts as a renewable resource through which Yan articulates his ideological and aesthetic vision, sustaining a critical engagement with ecological consciousness [13]. In *Dream of Ding Village*, for instance, the pond transformed into a “blood pond” through the washing of used blood bags, together with the unnatural croaking of frogs and the mutation of insects, vividly illustrates how the natural environment is foregrounded as a central locus of narrative and ethical inquiry. These ecological distortions manifest a chaotic era refracted through the prism of distorted human ethics and projected onto nature itself. Consequently, the environment functions less as a setting than as a photographic plate, revealing how external crises are internalized by the human spirit and subsequently “developed” into physical reality. Similarly, in the *Balou Mountain* narratives, the portrayal of local landscapes underpins a “utopian cognitive mapping,” demonstrating how the natural environment serves as a foregrounding device to construct both narrative space and ideological meaning.

Within the rural framework, the semantic domains associated with spatial narrative (H2, H1) play a pivotal role in delineating both the physical and psychological boundaries of the characters’ lived experiences. Recurring architectural motifs, specifically rooms, doors and courtyards, establish an isomorphic correspondence between visible space and invisible order. Here, the manipulation of thresholds, such as the opening and closing of doors, reifies social power dynamics, ethical stances, and psychological constraints. Through the spatial coding, physical settings function as external manifestations of the characters’ “inner causality.” These architectural features frequently coincide with moments of acute narrative tension, exposing the suppression of individual agency within the overarching socio-political structure. On a macro level, mythorealism constructs a narrative space wherein the trauma of ordinary people is articulated through an absurdity that paradoxically accentuates the reality of historical events [3]. Yan’s structural prioritization of space attests to the influence of spatial storytelling traditions characteristic of Western modernist literature [5,18,20].

In Yan’s works, the color *red* carries profound symbolic weight, functioning as a visual code that mediates between cultural surface and spiritual depth. While the color conventionally denotes auspiciousness in Chinese tradition, Yan employs it to construct a potent symbolic irony, juxtaposing these positive associations with narratives of suffering and punishment. In the novella *Gold Cave*, for instance, *red* emerges as a visceral cultural image that provokes cognitive dissonance [20]. Thus, a defining mechanism of mythorealism is the translation of spiritual experience into cultural codes: red blossoms, red silk, and bright red emotions do not merely mark narrative events but externalize individual yearnings for freedom, redemption, and honor into concrete visual imagery. Through the tension of these chromatic contrasts, the narrative guides readers to perceive ethical truths that linear causality alone cannot articulate. Accordingly, the imagery identified within this semantic domain serves as a vital vehicle for the symbolism, imbuing the text with stratified meanings that reveal the invisible cultural forces shaping the characters’ destinies.

Regarding the final pattern, supernatural elements underscore a defining stylistic feature of mythorealism, the deployment of “inner causality” to access the bedrock of spiritual reality. As Rojas and Fu [51] observe, Yan employs an interplay of distinct discursive modes, including biblical, mythological, and historiographical, to probe the depths of the human spirit while engaging critically with China’s socio-historical realities. It is within this domain that “inner causality” decisively supplants the “surface causal logic of everyday life.” In *The Four Books*, this principle is epitomized by The Child’s self-crucifixion and The Scholar’s revision of the Sisyphus myth. The Child invokes “Heaven” to abandon the logic of secular survival for a redemptive logic of the soul, while The Scholar’s inverted myth functions as a psychic projection articulating his spiritual alienation. Therefore, the narrative trajectory is governed not by external constraints, but by the characters’ introspective quest to rewrite the internal logic of their existence. These events defy empirical probability; rather, they serve as “mythorealist” projections of the intellectual psyche under historical contexts. Here, the supernatural operates as a structural device that transcends traditional realism to interrogate sin, redemption, and human nature. In essence, the dynamic crystallizes the relationship between mythorealism and reality, revealing a truth that is “non-existent” in the physical world yet “absolutely true” in the realm of the spirit [18].

Fundamentally, mythorealism constitutes a literary practice deeply rooted in Chinese reality yet significantly informed by twentieth-century Western modernism [5,18,20]. Within a corpus-based framework, the foregrounded stylistic features of Yan’s works encompass: a sustained engagement with rural culture under specific political conditions; the utilization of political discourse as a vehicle for both collective rhetoric and spiritual inquiry; the deployment of spatial narratives that map physical environments onto psychological terrains; the mobilization of color symbolism as a cultural code to convey ethical irony; and the suspension of linear causality, which embeds multiple discursive modes to penetrate the surface of historical reality and transmute the visible into ontological and ethical truth. The dynamic elucidates why the identified semantic patterns remain anchored in social reality while consistently gravitating toward spiritual interpretation. By prioritizing “inner causality” over empirical logic, mythorealism not only challenges conventional narrative forms but also offers a profound reflection on the contradictions of contemporary Chinese society [6]. As Rojas [52] observes, rather than positing a stance outside ideology, Yan’s practice interrogates the nexus of reality, ideology, and literary production, demonstrating that mythorealism functions as a sophisticated mode of historical commentary illuminating the interplay between narrative form and the deep, invisible currents of sociopolitical content.

## 6. Conclusion

The application of corpus stylistics to analyze Yan Lianke’s mythorealism presents a distinct methodological perspective, revealing new thematic and stylistic layers in his work. The analysis demonstrates how mythorealism manifests in English translation through five foregrounded semantic patterns: political discourse, spatial narrative, the natural environment, color symbolism, and supernatural elements. Collectively, these patterns constitute empirical evidence that Yan’s translated fiction consistently embodies the core hallmarks of mythorealism, constructing a stylistic architecture that reconfigures historical reality into allegorical critique. Furthermore, the study’s empirical findings vindicate the methodological strategy proposed at the outset. While the initial decision to utilize English translations was driven by the technical asymmetry of computational tools, the analysis confirms that this approach serves as an effective bridge to the source text’s semantic macrostructures. The validity is rigorously substantiated by the bilingual case study of *revolution*, which reveals that the translation maintains the original concept’s core collocational network, with divergences appearing as a reweighting of peripheral associations (i.e., the modulation of embodied versus institutional nuances). The evidence confirms that although the translator serves as a micro-level regulator adjusting stylistic intensity, the translation functions as a robust vessel for Yan’s essential thematic and narrative architecture.

Building on the present analysis, future research may fruitfully pursue two key avenues. First, while this study offers a synchronic overview of Yan’s translated oeuvre, it leaves the diachronic evolution of his style unmapped. A longitudinal investigation would therefore offer nuanced perspectives on how his mythorealist aesthetic has responded to shifting socio-political landscapes and artistic maturation. Second, the macro-level findings identified here provide a grounding for granular qualitative inquiry, inviting the use of parallel corpora for cross-linguistic comparison. Such an approach would allow for a rigorous examination of the translation strategies employed to render Yan’s unique narrative features, which are crucial for understanding the text’s cross-cultural reception. Taken together, integrating diachronic and bilingual methodologies represents a vital pathway for delineating how mythorealism transcends temporal and linguistic boundaries.

## Supporting information

S1 File Dataset metadata and Wmatrix outputs. (ZIP)
