# Supplementary figures and images for: Discovering mythorealism: A corpus stylistic analysis of Yan Lianke’s novels in English
Source: PLoS One. 2026 Feb 24;21(2):e0342696. doi: 10.1371/journal.pone.0342696 (PMC12931789; doi:10.1371/journal.pone.0342696)

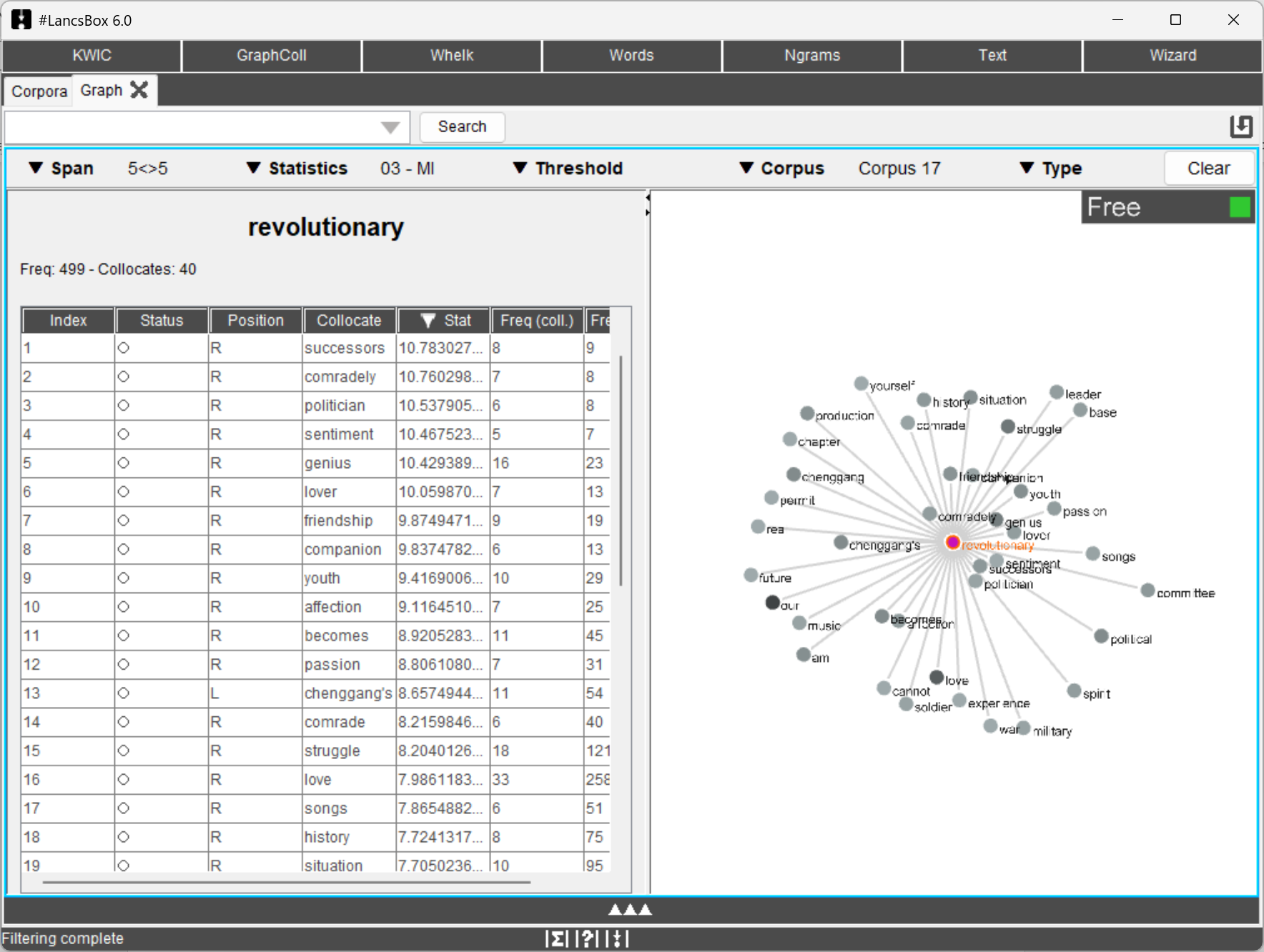

Supplement: S1 File — (ZIP) [file pone.0342696.s001.zip › S1_Dataset_Metadata_and_WmatrixOutputs/Collocates and concordance lines of keywords from excluded semantic domains/屏幕截图 2025-10-07 172310.png]

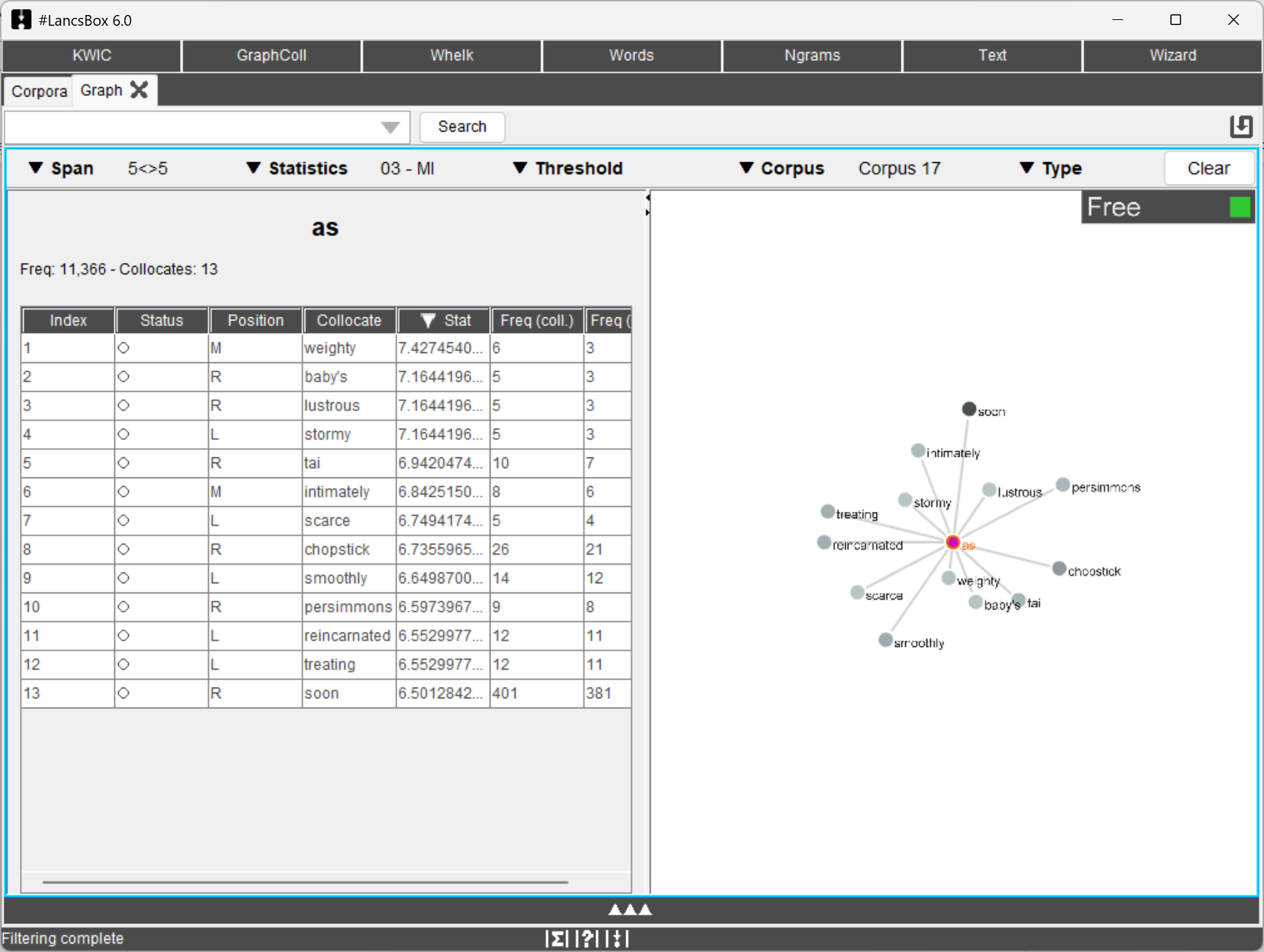

Supplement: S1 File — (ZIP) [file pone.0342696.s001.zip › S1_Dataset_Metadata_and_WmatrixOutputs/Collocates and concordance lines of keywords from excluded semantic domains/屏幕截图 2025-10-07 172433.png]

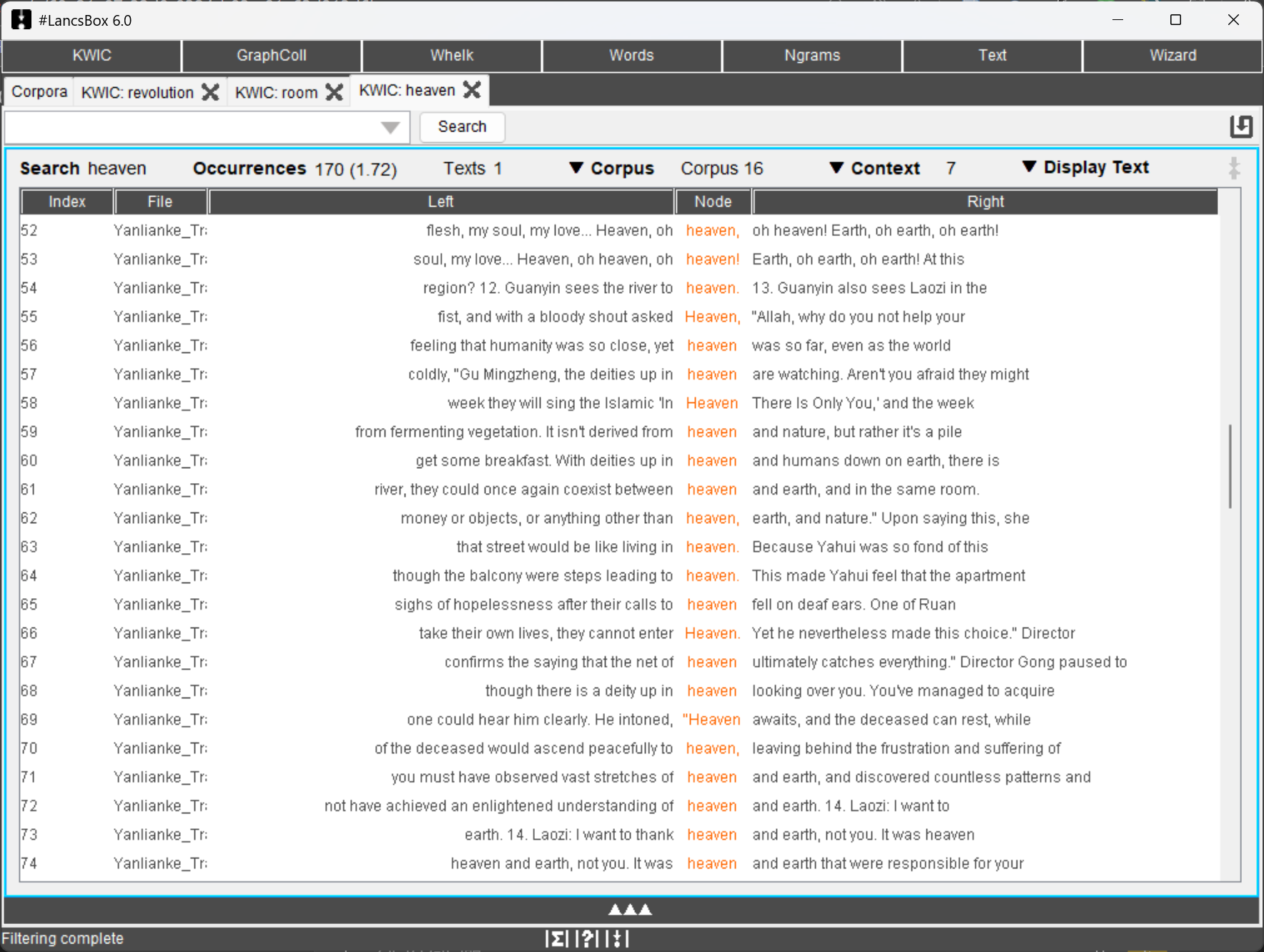

Supplement: S1 File — (ZIP) [file pone.0342696.s001.zip › S1_Dataset_Metadata_and_WmatrixOutputs/Retrieving concordance lines using the LancsBox software/Retrieving concordance lines containing “heaven” using the LancsBox software..png]

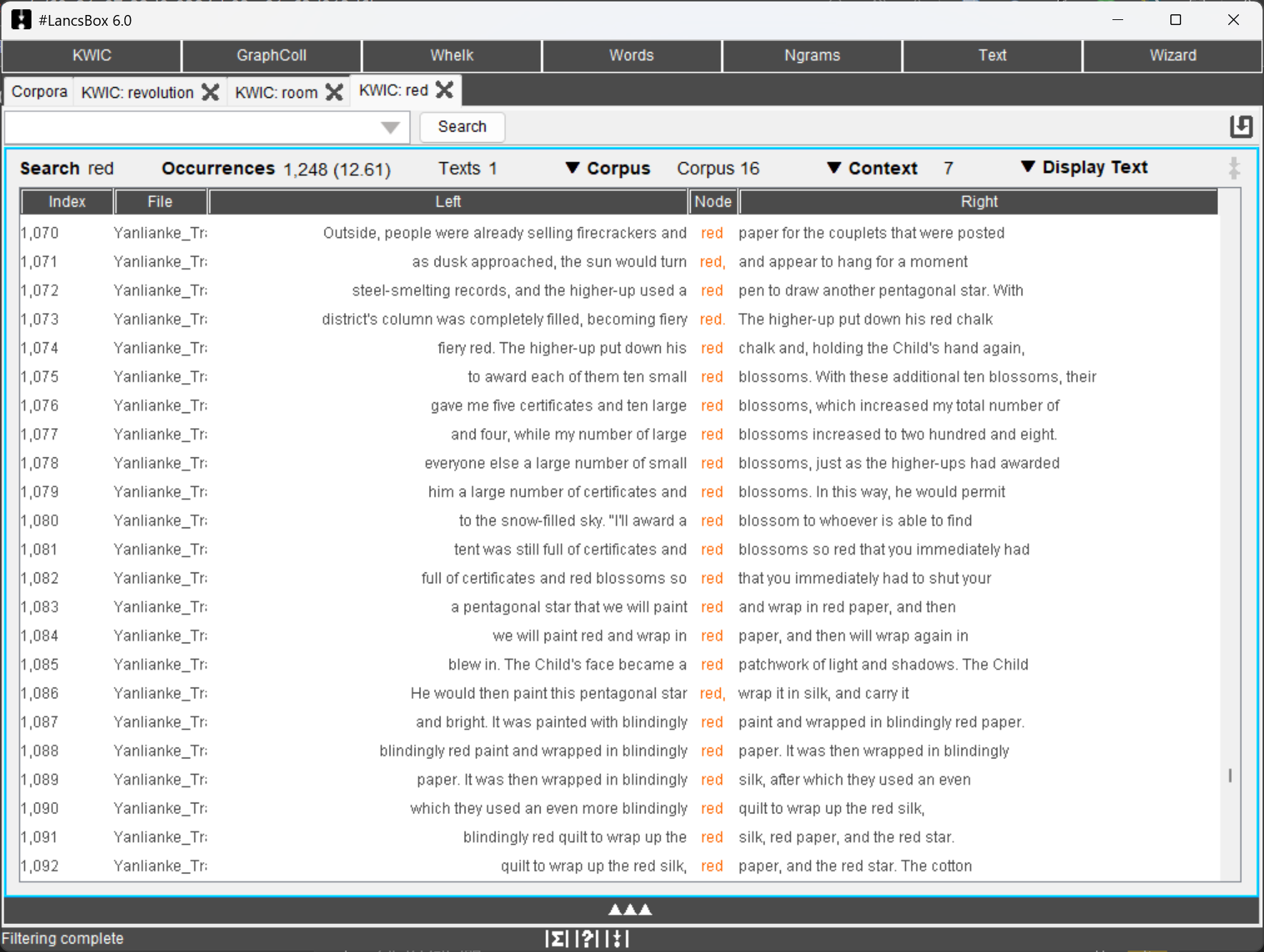

Supplement: S1 File — (ZIP) [file pone.0342696.s001.zip › S1_Dataset_Metadata_and_WmatrixOutputs/Retrieving concordance lines using the LancsBox software/Retrieving concordance lines containing “red” using the LancsBox software..png]

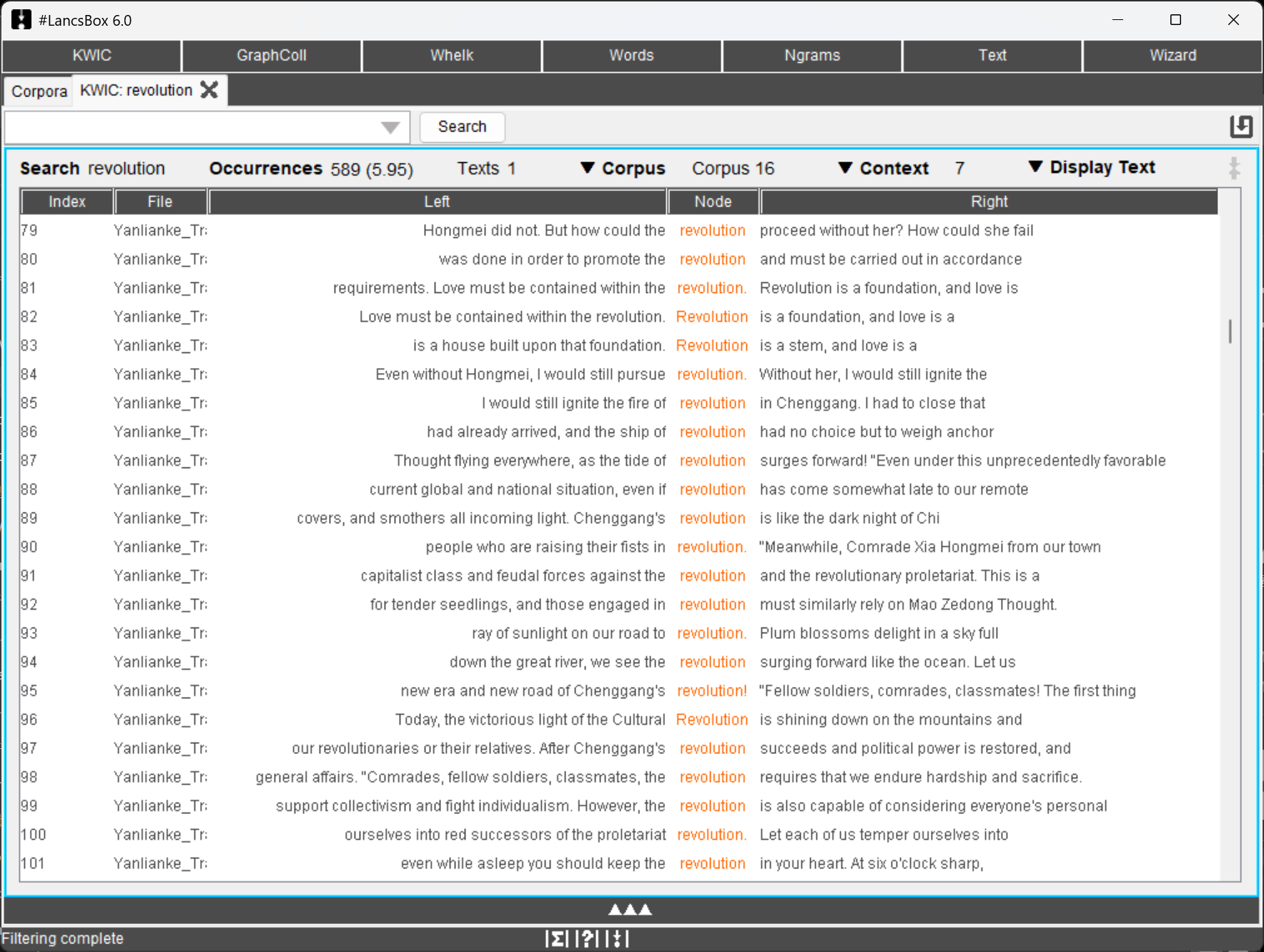

Supplement: S1 File — (ZIP) [file pone.0342696.s001.zip › S1_Dataset_Metadata_and_WmatrixOutputs/Retrieving concordance lines using the LancsBox software/Retrieving concordance lines containing “revolution” using the LancsBox software..png]

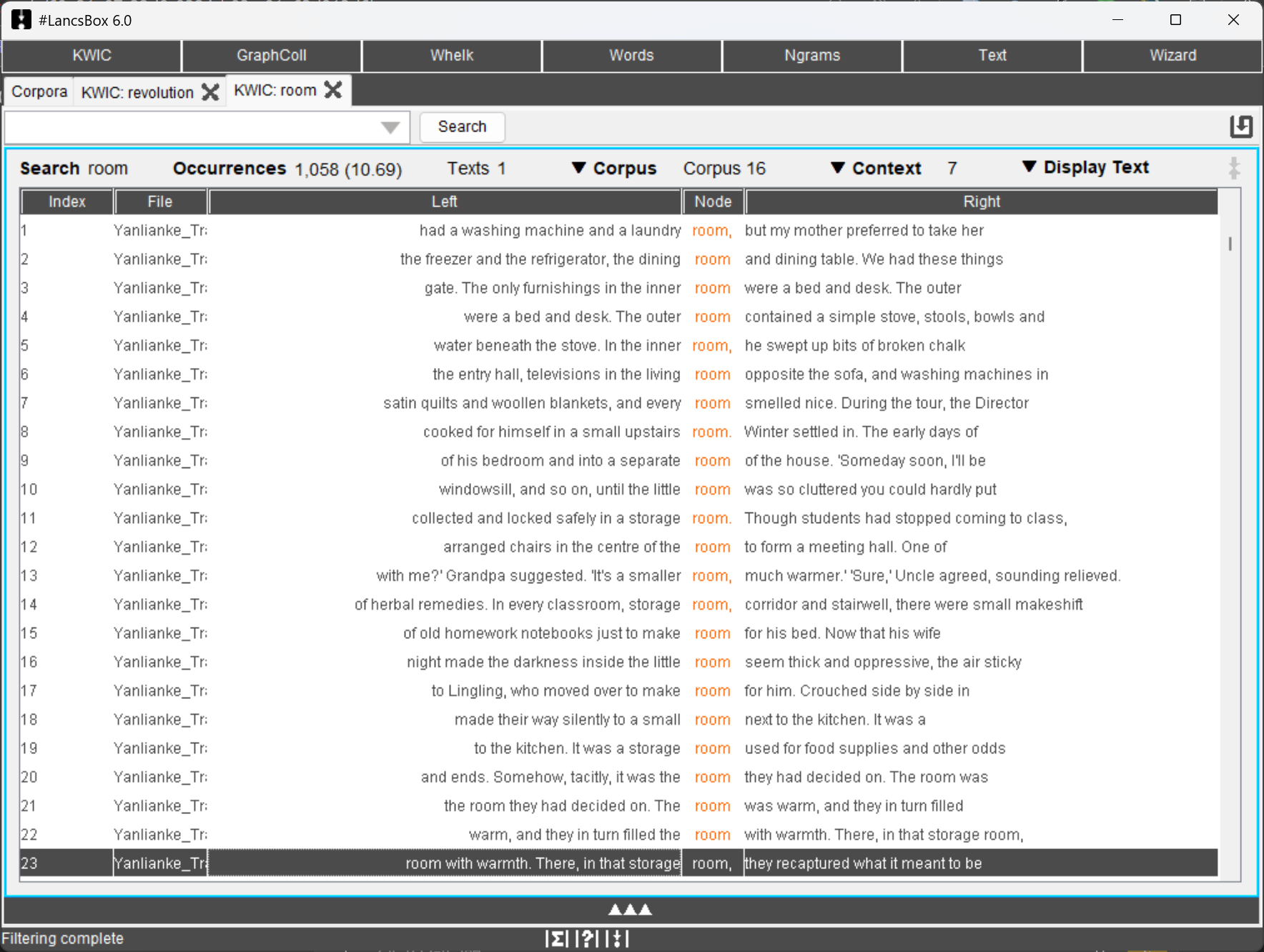

Supplement: S1 File — (ZIP) [file pone.0342696.s001.zip › S1_Dataset_Metadata_and_WmatrixOutputs/Retrieving concordance lines using the LancsBox software/Retrieving concordance lines containing “room” using the LancsBox software..png]
